# Prediction of opioid-related outcomes in a medicaid surgical population: Evidence to guide postoperative opiate therapy and monitoring

**DOI:** 10.1371/journal.pcbi.1011376

**Published:** 2023-08-14

**Authors:** Oualid El Hajouji, Ran S. Sun, Alban Zammit, Keith Humphreys, Steven M. Asch, Ian Carroll, Catherine M. Curtin, Tina Hernandez-Boussard

**Affiliations:** 1 Department of Medicine, Stanford University, Stanford California, United States of America; 2 Institute for Computational & Mathematical Engineering, Stanford University, Stanford California, United States of America; 3 Center for Innovation to Implementation, Palo Alto Veterans Affairs Healthcare System, Palo Alto California, United States of America; 4 Department of Psychiatry and the Behavioral Sciences, Stanford University, Stanford California, United States of America; 5 Department of Anesthesiology, Perioperative, and Pain Medicine, Stanford University, Stanford California, United States of America; 6 Department of Surgery, VA Palo Alto Health Care System, Menlo Park California, United States of America; 7 Department of Surgery, Stanford University, Stanford California, United States of America; 8 Department of Biomedical Data Science, Stanford University, Stanford California, United States of America; University of Washington, UNITED STATES

## Abstract

**Background:**

Treatment of surgical pain is a common reason for opioid prescriptions. Being able to predict which patients are at risk for opioid abuse, dependence, and overdose (opioid-related adverse outcomes [OR-AE]) could help physicians make safer prescription decisions. We aimed to develop a machine-learning algorithm to predict the risk of OR-AE following surgery using Medicaid data with external validation across states.

**Methods:**

Five machine learning models were developed and validated across seven US states (90–10 data split). The model output was the risk of OR-AE 6-months following surgery. The models were evaluated using standard metrics and area under the receiver operating characteristic curve (AUC) was used for model comparison. We assessed calibration for the top performing model and generated bootstrap estimations for standard deviations. Decision curves were generated for the top-performing model and logistic regression.

**Results:**

We evaluated 96,974 surgical patients aged 15 and 64. During the 6-month period following surgery, 10,464 (10.8%) patients had an OR-AE. Outcome rates were significantly higher for patients with depression (17.5%), diabetes (13.1%) or obesity (11.1%). The random forest model achieved the best predictive performance (AUC: 0.877; F1-score: 0.57; recall: 0.69; precision:0.48). An opioid disorder diagnosis prior to surgery was the most important feature for the model, which was well calibrated and had good discrimination.

**Conclusions:**

A machine learning models to predict risk of OR-AE following surgery performed well in external validation. This work could be used to assist pain management following surgery for Medicaid beneficiaries and supports a precision medicine approach to opioid prescribing.

## Author summary

The opioid epidemic continues in the United States and prescription opioids contribute to opioid-related overdose deaths. The study aimed to develop a machine-learning algorithm to predict the risk of opioid-related adverse outcomes (OR-AE) following surgery using Medicaid data with external validation across seven US states. This retrospective study of 96,974 patients presents seven machine learning models to predict opioid-related adverse outcomes (OAO) following surgery. The best predictive performance was achieved by the random forest model, with an AUC of 0.877. Patients with depression, diabetes, or obesity had higher rates of OR-AE. The model was well calibrated and had good discrimination, with an opioid disorder diagnosis prior to surgery being the most important feature. This work provides evidence that an automated risk prediction can potentially guide postoperative opiate therapy and monitoring.

## Introduction

The risks of opioids including dependence, misuse and overdose death are now well documented. In the United States, the opioid epidemic is a national public health emergency, with recent estimates suggesting that more than 10 million Americans aged 12 or older misused opioids in 2020 [1]. Prescription opioids are a major contributor to misuse, which can lead to addiction and death. Drug overdose deaths involving prescription opioids rose from 3,442 in 1999 to 16,416 in 2020 [2].

Treatment of surgical pain is the second most common reason patients receive opioids, and thus is an important avenue of exposure to opioid-related risks [3,4]. Postoperative pain management attempts to balance opioid-facilitated analgesia against the inherent risks of opioids. Inadequate pain management besides causing human suffering, increases risk of complications including the development of chronic pain [5]. However, the receipt of prescription opioids after surgery is associated with increased risks of long-term opioid use disorders [6–8]. Overprescribing can also lead to opioid diversion with the excess opioids [9]. A recent meta-analysis suggests that surgical procedures are associated with nearly 7% of patients experiencing persistent opioid use after surgery [10]. Understanding how to provide adequate pain management and mitigate the risk of developing opioid use disorders remains a top question in the surgical field.

The Medicaid population presents unique challenges in perioperative pain management because multiple stressors can complicate their pain outcomes [11–13]. For example, opioid use disorder (well known to adversely impact pain outcomes) is higher among Medicaid beneficiaries [14]. Among the two million nonelderly US adults with opioid use disorder, nearly 40% were covered by Medicaid [15]. Increased socioeconomic stressors and pre-existing opioid exposures makes perioperative pain management and use of opioids more fraught in the Medicaid population.

The prevalence of opioid-related adverse outcomes (OAO) in the Medicaid population is not well-documented. Predicting risks within the covered population can help state Medicaid programs implement interventions targeting individuals at high-risk for adverse opioid-related outcomes and to promote precision medicine. The aim of this paper is to develop a risk score for Medicaid patients who are at high risk for opioid abuse, dependence and overdose following surgery. This score can be used to identify patients at risk who might benefit from alternative pain management modalities or need additional monitoring and support. In addition, understanding the risk of OAO in vulnerable populations (i.e., patients with depression, or previous substance abuse can inform prescribing practices by clinicians to these higher-risk patients.

## Methods

### Ethics statement

We received the HIPAA patient waiver allowing us to access and use the patient’s medical records for research purposes. This study received the approval from the Stanford University’s Institutional Review Board (IRB), #47644.

### Data source

This retrospective cohort study consisted of de-identified enrollee data obtained from Medicaid enrollment and claims provided by the Digital Health Cooperative Research Center. Data collected were from three western states, two midwestern states and two southeastern states between January 2016 and June 2020. Two states provide data on the entire Medicaid population including both the managed care plan and the fee-for-service patients. Four states provide data from a managed care plan and one state had only fee-for-service Medicaid beneficiaries. Data were reported according to MINIMAR guidelines [16].

### Cohort selection

We included Medicaid enrollees who underwent one of 19 common types of surgery as identified by the International Classification of Diseases 9 and 10 codes and Current Procedural Terminology (CPT) codes. We categorized surgeries using the Clinical Classifications Software for Services and Procedures (CCS-Services and Procedures). (S1 Table). We included the most common major surgeries but excluded those that would likely have a low pain profile such as cataracts, breast biopsies, etc. We excluded individuals who underwent more than one surgery to ensure that any opioid event (e.g., prescription, opioid disorder diagnosis) is not related to another surgery. We included enrollees with continuous Medicaid coverage for at least 6 months prior and 12 months after the surgery event. This ensured data observability and a complete capture of persons’ characteristics and outcomes needed for prediction. We excluded enrollees who were 65 years of age and older due to their dual eligibility for Medicare, for which we had no claim data. The starting age of inclusion was 15 years, because prescription and illicit opioid use among the 15–19 age group is high [17]. Opioid prescriptions were identified using RxNorm identifiers from the *Unified Medical Language* system (opioid prescriptions are listed in Appendix A). Opioid prescriptions were also converted to Morphine Milligram Equivalent (MME) associated following CDC recommendations [18].

### Feature construction

Our primary outcome was a binary indicator of whether a person had opioid abuse, dependence, or an overdose event (opioid-related adverse outcomes) during the 6-month period after surgery. OAO were identified by the *International Classification of Diseases*, *9th Revision* (ICD-9-CM) codes and the *International Classification of Diseases*, *10th Revision* (ICD-10-CM) codes summarized in S2 Table. We did not separate these adverse outcomes due to the limited event incidence of opioid overdose. Our secondary outcome was persistent opioid use, a binary variable indicating whether a patient had a new opioid prescription between 3 and 6 months after discharge from surgery [4].

Sociodemographic features, including age, gender and urbanization status based on an enrollee’s zip code were included. These variables were in a standard format and therefore comparable across states. We did not include race and ethnicity features due to lack of data availability. Based on the diagnosis codes available in the claims during the 6 months pre-surgery, we extracted 544 diagnosis features corresponding to the Clinical Classifications Software Refined (CCSR) ICD-10-CM diagnosis categories (S3 Table) and used the CCSR to identify diabetic, obese and depressed populations [19]. Similarly, 326 procedure features were extracted corresponding to the CCSR ICD-10-PCS procedure categories. We excluded those that were reported in less than 2% of patients. With a threshold of less than 2%, we are able to exclude features with very low prevalence, as they may not have enough variation to be useful for prediction and including them in the model may actually decrease its performance. This feature selection process resulted in 227 diagnosis features and 38 procedure features used as model inputs. In addition, type of surgery was used as a feature. Based on a combination of opioid and non-opioid prescription claims 6-months preceding surgery, a total of 46 prescription features were extracted. Patients who were not exposed to opioids during the 6 months period before surgery were classified as opioid naïve. Morphine milligram equivalent (MME) were computed using the CDC formula, as previously described in detail.^18^ All features are described in S4 Table.

### Model development and construction

The primary goal was to predict 6-month postoperative risk of an OAO and the secondary goal was to predict risk of persistent opioid use. The steps for both prediction goals were the same. Medicaid data from 7 states were combined and then divided randomly into a training set comprising 90% of the data and a test set comprising 10%. We randomly sampled (stratifying by outcome) a training set from the cohort consisting of 90% of the data, the rest of the data being used as a testing set. Due to class imbalance, the pipelines consisted of a step of median imputation for missing values (for age, gender and urbanization status features), a step of standardization for numeric features, a step of minority class over-sampling with ratio 0.3, a step of majority class under-sampling with ratio 0.7 (resulting in a 41% outcome rate), followed by the machine learning model. We built our prediction models using multiple machine learning as well as deep learning methods: logistic regression and its variants Ridge, Lasso and Elastic Net, random forest, extreme gradient boosted trees (xgboost) and a deep neural network approach. For all pipelines, hyperparameters were selected using a grid search with 5-fold cross-validation on the training data, optimizing for area under the receiver operating characteristic curve (AUC). Hyperparameters are reported in S5 Table. In practice, identifying all patients with high risk of having OAO is desired, thus we favor recall over precision in the precision-recall trade-off.

### Model evaluation

We ran the models on 5 subsets of the held-out test set and computed means and standard deviations of several metrics obtained on each subset. Evaluation was done by comparing the means of these metrics. We reported AUC, precision, recall and F1 score to show the prediction ability. We prioritized AUC for comparison since it is threshold-independent, so that it measures the overall prediction ability of the model. The other metrics measure a snapshot of the model in the precision-recall trade-off. A precision-recall curve was generated for the best performing model for the primary outcome prediction.

To assess the calibration of the top performing model, we conducted several analyses. First, we calculated the calibration intercept and slope and generated calibration curves specific to each population. Additionally, we generated bootstrapped estimates of standard deviations for the overall calibration curve. The calibration intercept assesses whether risks are overestimated (intercept <0) or underestimated (intercept >0). An extreme slope leads to underestimation of low risks and overestimation of high risks, whereas a moderate slope results in overestimation of low risks and underestimation of high risks [20]. We used these metrics to evaluate the performance of our model. Finally, we generated overall decision curves for both the top performing model and the logistic regression to visualize the potential clinical impact of using each model. By presenting these curves, we aimed to provide clinicians with an intuitive way to understand the potential benefits and harms of using each model.

### Feature importance

For the primary outcome, to better understand the prediction and how different features impact the model result, we generated the top 20 important features using Shapley-value feature importance obtained from the best performing model [21]. We applied hierarchical clustering to the original features based on their Spearman correlations so that correlated features can be grouped in clusters. We then chose one feature per cluster and ran the feature importance analysis based on those newly selected features. Without such a subset selection procedure, the model could not be interpreted accurately because of feature correlations. Indeed, the estimated importance of a feature correlated with a lot of other features is an underestimation of the actual importance.

### Statistical analysis

Descriptive statistics were used for the entire sample as well as for individuals with OAO during the 6-month period after surgery. We measured the bivariate association of the categorical variables with the primary outcome using the chi-squared test of independence and we measured the association of age to the primary outcome using Welch’s t-test. Additionally, we evaluated the primary outcome and average discharge MME within the diabetic, obese and depressed population subsets of the cohort overall and across all years, using a t-test for regression slope comparison with the control population. Statistical significance was set as 2-tailed p<0.05.

## Results

### Cohort characteristics

**Fig 1:**
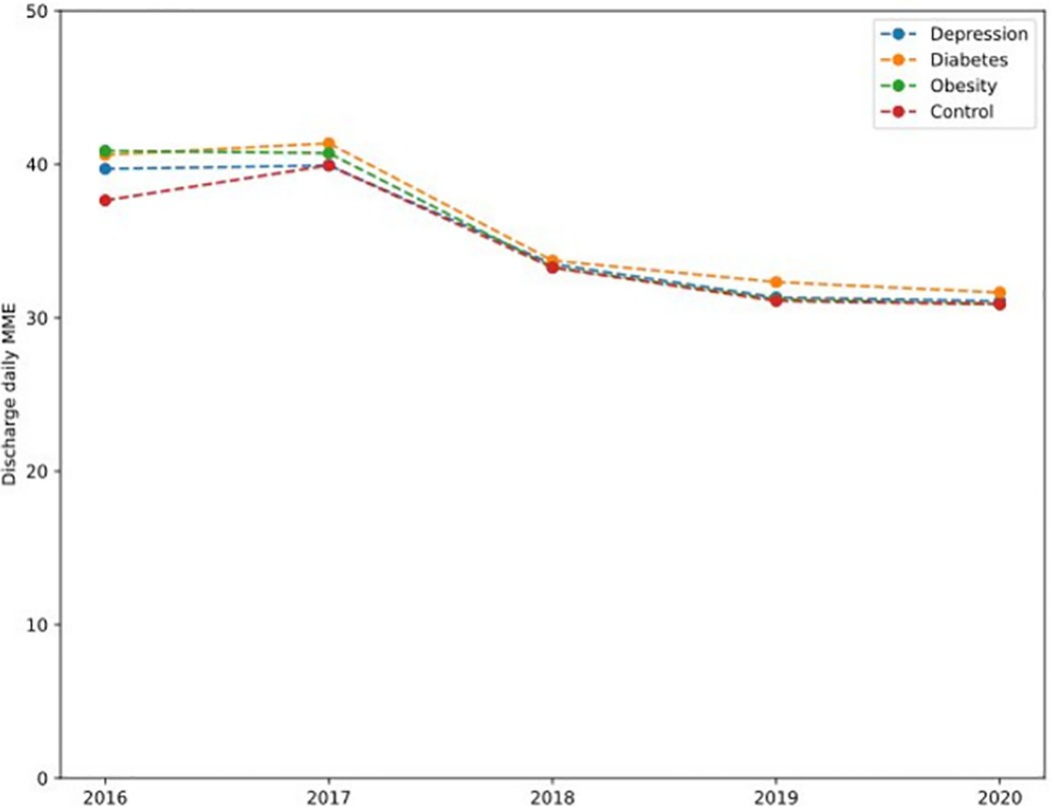
Discharge daily MMEs for vulnerable populations across years of surgery.

**Fig 2:**
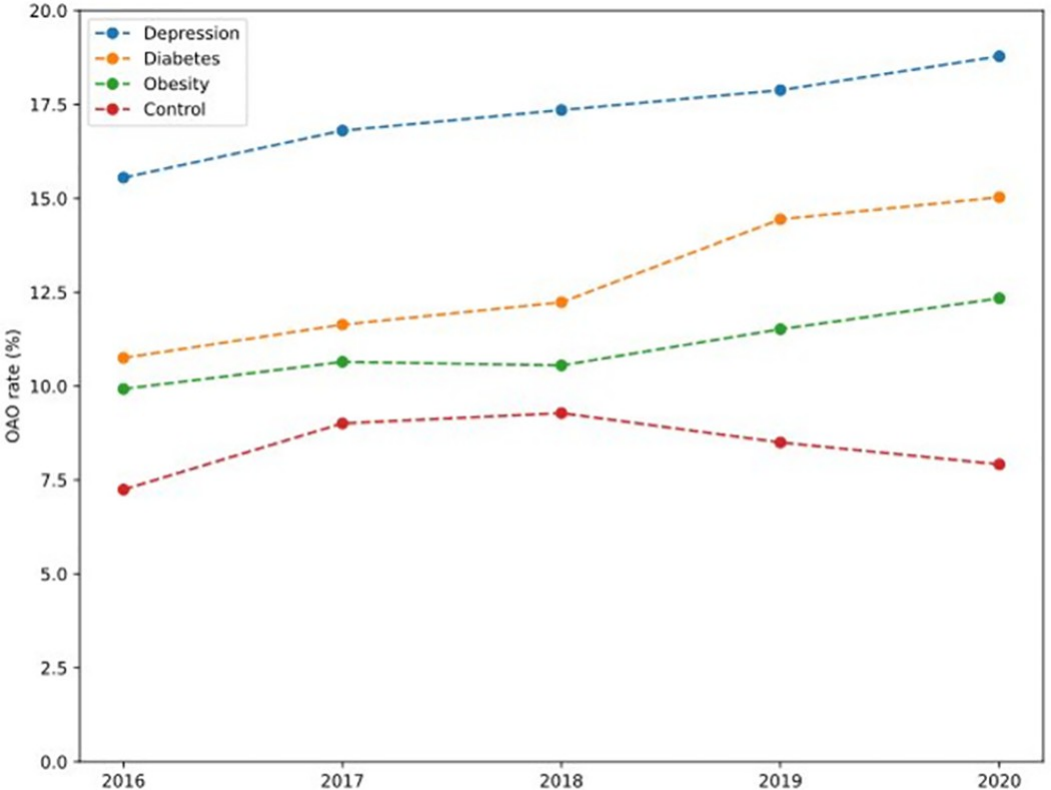
Opioid Outcomes for vulnerable populations across years of surgery.

**Fig 3:**
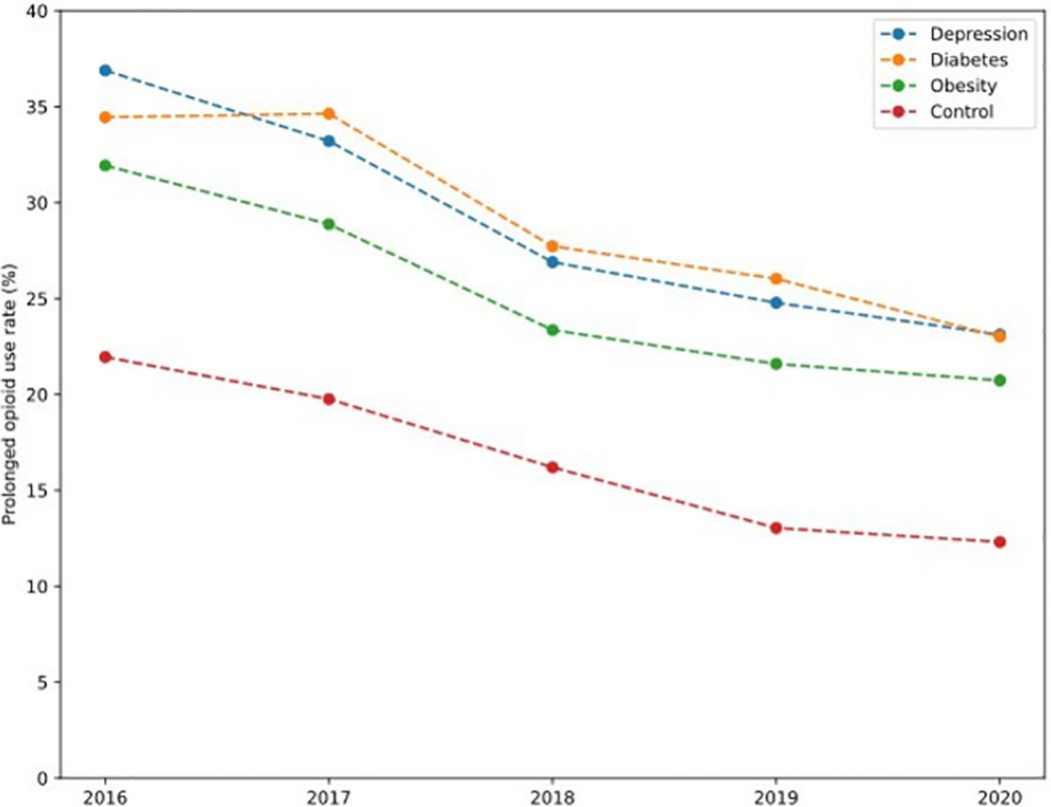
Prolonged Opioid Use for vulnerable populations across years of surgery.

A total of 96,974 enrollees were included in the study (S1 Fig). Table 1 presents the characteristics of the study cohort, overall and by outcome category. The cohort was on average 40.5 (SD 13.5) years old with 69.4% female. Opioid naive patients account for 66.2% of all enrollees. A higher proportion of males had an opioid-related adverse outcome compared to females (12.2% vs. 10.2%, p<0.001). By 6-month post-surgery, 10,018 (10.3%) patients had opioid abuse or dependence and 908 (0.9%) had at least one overdose event, totaling a 10.8% OAO rate. 20,075 (20.7%) patients had persistent opioid use. Patients with diabetes, depression, and obesity had a higher rate of OAO compared to non-vulnerable patients. We report the evolution, between 2016 and 2020, of the discharge daily MME (Fig 1), persistent opioid use rate (Fig 2) and OAO rate (Fig 3) for these populations, as well as the control population. The average daily MME discharge prescription across the populations was not significantly different. As for persistent opioid use rates, those are significantly higher for patients with diabetes, depression, and obesity than for the control population (p<0.001), but all the rates decrease between 2016 and 2020. The difference of trend was statistically significant for the depressed (p = 0.012) and diabetic (p = 0.009) populations. The year-on-year changes of OAO rates are 0.75 percentage points for depression (p = 0.002), 1.14 percentage points for diabetes (p = 0.004) and 0.57 percentage points for obesity (p = 0.010).

**Table 1:** Patient demographics stratified by opioid adverse outcome status*.

Variable		Total	No Opioids Adverse Outcome	Opioids Adverse Outcome
**N**		96974	86510 (89.2)	10464 (10.8)
**Age, mean (SD)**		40.5 (13.5)	40.0 (13.7)	44.0 (11.5)
**Gender, n (%)**	**Female**	66860 (69.4)	60027 (89.8)	6833 (10.2)
	**Male**	29509 (30.6)	25910 (87.8)	3599 (12.2)
**Opioid Naive, n (%)**		64180 (66.2)	59448 (92.6)	4732 (7.4)
**State, n (%)**	**State 1**	38922 (40.1)	34891 (89.6)	4031 (10.4)
	**State 2**	38717 (39.9)	34552 (89.2)	4165 (10.8)
	**State 3**	7980 (8.2)	6906 (86.5)	1074 (13.5)
	**State 4**	4731 (4.9)	3937 (83.2)	794 (16.8)
	**State 5**	4164 (4.3)	3917 (94.1)	247 (5.9)
	**State 6**	1487 (1.5)	1411 (94.9)	76 (5.1)
	**State 7**	973 (1.0)	896 (92.1)	77 (7.9)
**Surgery Year, n (%)**	**2016**	11131 (11.5)	10101 (90.7)	1030 (9.3)
	**2017**	21524 (22.2)	19263 (89.5)	2261 (10.5)
	**2018**	21674 (22.4)	19271 (88.9)	2403 (11.1)
	**2019**	28161 (29.0)	25003 (88.8)	3158 (11.2)
	**2020**	14484 (14.9)	12872 (88.9)	1612 (11.1)

*All p-values are <0.001

### Model performance

The results for the models for both outcomes are reported in Table 2, S6 and S7 Tables. The random forest model achieved the highest score for all metrics: 0.877 for AUC, 0.57 for F1 score, 0.69 for recall and 0.48 for precision. A precision-recall curve for the random forest model is displayed in S2 Fig. For the persistent opioid use prediction, the F1 scores are overall comparable to the best F1 score for OAO prediction.

**Table 2:** Best models’ performance metrics for opioid adverse outcome prediction and persistent opioid use prediction.

Outcome	AUC	F1	Recall	Precision
**Opioid adverse outcome (OAO)**	0.877	0.57	0.69	0.48
**Persistent opioid use**	0.819	0.59	0.52	0.68

**Fig 4:**
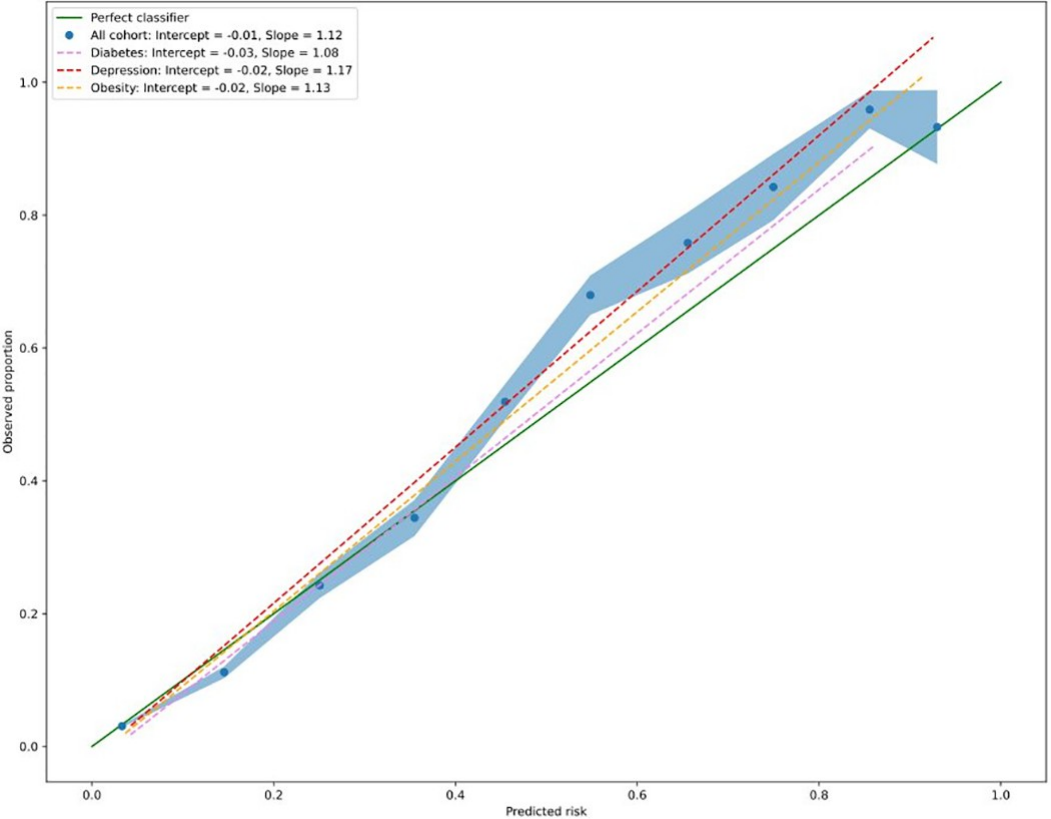
Summary figure with overall calibration curves.

**Fig 5:**
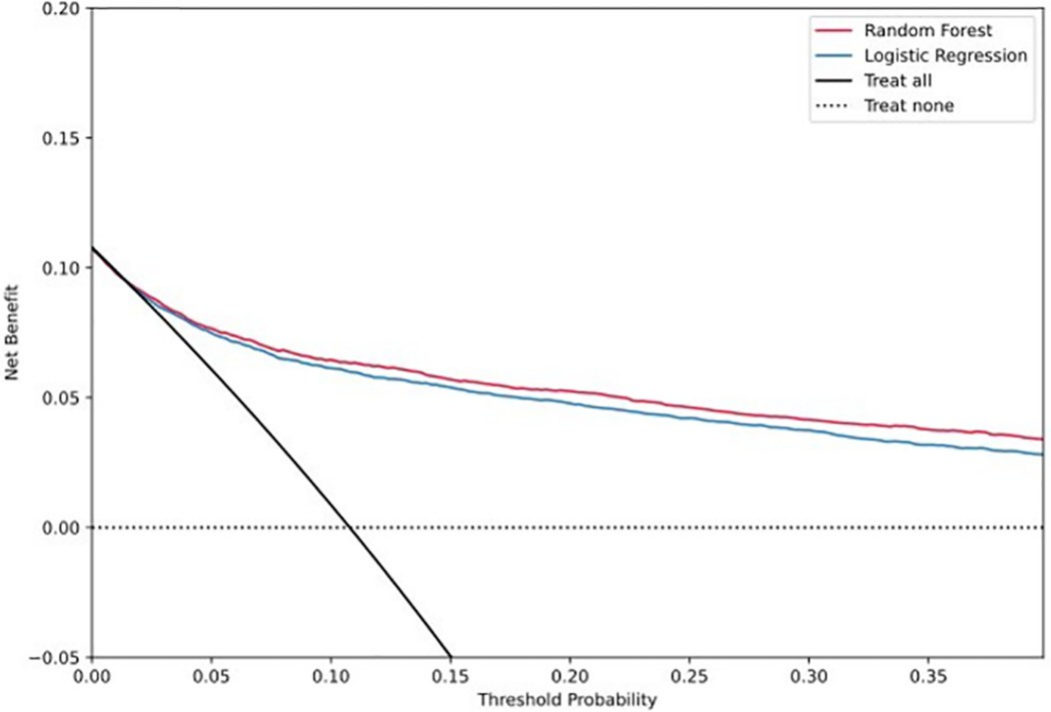
Decision curves for the top performing risk prediction model.

The random forest model had an overall calibration intercept of -0.01 and a slope of 1.12. Risk estimates were slightly underestimated (Figs 4 and 5). Calibration for the diabetes subpopulation was marginally better than the overall cohort. The random forest model had improved clinical utility to select patients at high risk of OAO compared to the logistic regression, as defined by the decision curve analysis; the higher the curve, the better the clinical utility at the chosen risk threshold.

### Model specification

**Fig 6:**
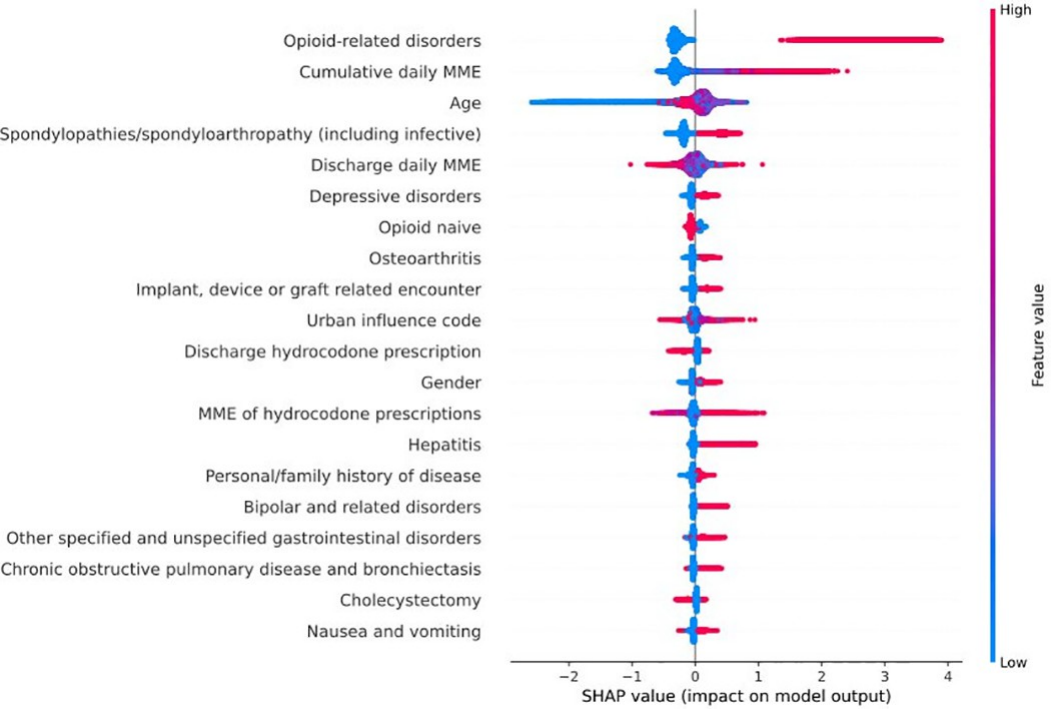
Shapley-value feature importance for top 20 features.

The top 20 features obtained from the Shapley-value method are reported in Fig 6. The most important feature associated with a higher likelihood of OAO is diagnosis of opioid-related disorders (pre-surgery). The second most important feature is cumulative daily MME (pre-surgery). Some factors were complex, influencing different populations in different manners. For example, a high discharge daily MME can lead both to predicting OAO and predicting no OAO. For the MME of hydrocodone prescriptions, most high values lead to OAO, but some high values are associated with a prediction of no OAO.

Age is the third most important feature, with young patients having less adverse outcomes. Gender is also part of the important features; males being more often associated with OAO. Urban influence code is also identified as one of the most important features: rural regions were predictive of both OAO and no OAO.

## Discussion

In this study we found more than 10% of Medicaid surgical patients were diagnosed with opioid abuse, dependence, or overdose after surgery, and more than 20% had persistent opioid use—requesting a new opioid prescription 3–6 months after surgery. We developed a machine learning approach to predict postoperative OAO in the Medicaid population. The models were reasonably calibrated, had good discrimination, and identified key features associated with OAO. The random forest model was best to distinguish between low and high-risk patients and thus had the highest potential clinical utility. If confirmed, these models could identify patients with a high risk of an OAO *prior* to surgery, increasing the care systems ability to change postoperative pain management among these high-risk patients to improve care outcomes, reduce unnecessary costs, and potentially reduce opioid misuse among this vulnerable population.

The models developed to predict OAO performed well, with AUC over 0.8. Previous opioid exposures (i.e., diagnosed opioid use disorder or high cumulative MME 6 months prior to surgery) were the strongest features associated with OAO. Although this is consistent with available literature,^3,8^ the machine learning approach builds on this evidence to generate a risk score for OAO in Medicaid patients that can be used prior to surgery to improve patient outcomes. Importantly, the machine learning model highlights a combination of features predicting adverse outcomes far better than any one individual feature.

Other diagnoses also put the patients at higher risk for OAO. A prior history of diabetes, depression, and obesity increased the risk by 2-fold. Moreover, we found that OAO rates increased across the study period in all vulnerable populations whereas the rest of the population had decreasing OAO. There are several potential explanations for this finding. First, these populations have increased healthcare needs and are at higher risk of adverse results after surgery that might require opiates [22–25]. Second, many of these patients are taking medications or have metabolic derangements that can complicate perioperative pain management [26,27]. This medical complexity could impact their pain management and opioid risk. These findings highlight how a machine learning approach can inform a practitioners approach pain management in unique populations and identify those that would most benefit from either increased monitoring, increased patient education, or reduced opioid dosing to improve outcomes, congruent with precision medicine initiatives.

Precision medicine considers the many variables that could affect the individual patient’s course to more accurately match their needs to therapy. The importance of these complex relationships is demonstrated by the machine learning models from this study, which are trained on 316 features. Machine learning gives a more nuanced picture than a simple binary outcome of a linear regression model. Models like random forests capture highly non-linear interactions of features and can take advantage of those interactions to predict the outcome. Therefore, one feature can have a positive influence on the outcome for some patients and a negative influence for others, depending on the feature’s complex interactions with other features. Shapley values provide a framework to explain the impacts on model outcomes of features while at the same time ranking features by importance. One example of this complexity was discharge daily MME, which is both a positive and negative predictor of OAO. This result means that discharge daily MME is important in pain response to surgery, but other factors importantly interact with discharge daily MME, influencing the outcomes.

There are several limitations to this study. First claims data do not provide functional measures, such as pain scores. However, we use a combination of prescriptions, encounters, and ICD codes to capture the patient care episode. Second, ICD codes for opioid abuse or overdose may be under-reported and do not distinguish between type of opioids, thus we cannot examine problems with prescribed opioid vs. illicit opioids. This likely means that models would perform better if there was less subjectivity in the outcomes. Finally, this study is based on data from seven states, therefore the results may not be generalizable to the entire Medicaid population. However, the data sets we have included are state-wide, geographically diverse and represent millions of Medicaid recipients. This allowed us to test the generalizability and robustness of our models across different states that were geographically diverse and had varying in demographic and socioeconomic characteristics. This allowed us to test the performance of our models in a range of different contexts and to assess their ability to generalize to new settings.

In this study, we characterize OAO following surgery using Medicaid administrative data and highlight the increased rates of these events in vulnerable populations. We present a risk score developed on pre-operative data that has good discrimination and calibration, which could allow one to identify patients at high risk of OAO prior to surgery and allow for either tailored pain management or increased monitoring following surgery. The approach highlights known prediction factors but also leverages the combination of interactions between variables to improve patient discrimination. Developing a risk tool flagging patients at high risk for developing OAO in clinicians’ workflow is crucial for optimizing treatment options and improving patient outcomes, particularly among vulnerable populations.

## Supporting information

S1 Table Type of surgical procedures included in the study with corresponding CCS procedure category. (DOCX)

S2 Table Diagnosis Codes for Opioid Abuse, Dependence and Overdose from International Classification of Diseases (ICD) 9th and 10th Revision. (DOCX)

S3 Table CCSR diagnosis categories for the 3 vulnerable populations. (DOCX)

S4 Table Description of features. (DOCX)

S5 Table Hyperparameters found by grid search with 5-fold cross-validation (Python 3.6.9). (DOCX)

S6 Table Model performance metrics for opioid abuse/dependence/overdose prediction. (DOCX)

S7 Table Model performance metrics for persistent opioid use prediction. (DOCX)

S1 Fig Flowchart for the study cohort. (DOCX)

S2 Fig Precision-recall curve for random forest model. (DOCX)
