# Supplementary material for: Prediction of opioid-related outcomes in a medicaid surgical population: Evidence to guide postoperative opiate therapy and monitoring
Source: PLoS Comput Biol. 2023 Aug 14;19(8):e1011376. doi: 10.1371/journal.pcbi.1011376 (PMC10449152; doi:10.1371/journal.pcbi.1011376)
Supplement: S1 Table — (DOCX) [file pcbi.1011376.s001.docx]

## sTable 1: Type of surgical procedures included in the study with corresponding CCS procedure category

| **Surgery types** | **Multi-Level CCS - Procedures** |
| --- | --- |
| Appendectomy | 9.12 |
| Coronary artery bypass graft (CABG) | 7.2 |
| Colorectal resection | 9.1 |
| Distal radius fracture* | 14.3 |
| Excision; lysis peritoneal adhesions | 9.22 |
| Hysterectomy; abdominal and vaginal | 12.5 |
| Inguinal and femoral hernia repair | 9.17 |
| Knee replacement* | 14.7 |
| Oophorectomy; unilateral and bilateral | 12.1 |
| Other hand* | 14.15 |
| Partial excision bone | 14.1 |
| Spinal fusion | 14.11 |
| Treatment; fracture or dislocation of hip and femur* | 14.3 |
| Treatment; fracture or dislocation of lower extremity* | 14.3 |
| Cholecystectomy and common duct exploration | 9.16 |
| Laminectomy; excision intervertebral disc | 1.3 |
| Mastectomy | 3.3 |
| Open prostatectomy | 11.2 |
| Thoracotomy* | 6.8 |

Abbreviations: CCS, Clinical Classifications Software.

* Type of surgery corresponding to a subset of CCS procedure category.
