# Supplementary material for: Prediction of opioid-related outcomes in a medicaid surgical population: Evidence to guide postoperative opiate therapy and monitoring
Source: PLoS Comput Biol. 2023 Aug 14;19(8):e1011376. doi: 10.1371/journal.pcbi.1011376 (PMC10449152; doi:10.1371/journal.pcbi.1011376)
Supplement: S2 Table — (DOCX) [file pcbi.1011376.s002.docx]

## sTable 2: Diagnosis Codes for Opioid Abuse, Dependence and Overdose from International Classification of Diseases (ICD) 9th and 10th Revision

| **Version** | **Condition** | **Code** | **Code Description** |
| --- | --- | --- | --- |
| ICD10 | opioid abuse | F11.1 | Opioid abuse |
| ICD10 | opioid abuse | F11.120 | Opioid abuse with intoxication, uncomplicated |
| ICD10 | opioid abuse | F11.121 | Opioid abuse with intoxication delirium |
| ICD10 | opioid abuse | F11.129 | Opioid abuse with intoxication, unspecified |
| ICD10 | opioid abuse | F11.15 | Opioid abuse with opioid-induced psychotic disorder |
| ICD10 | opioid abuse | F11.150 | Opioid abuse with opioid-induced psychotic disorder with delusions |
| ICD10 | opioid abuse | F11.188 | Opioid abuse with other opioid-induced disorder |
| ICD10 | opioid dependence | F11.21 | Opioid dependence, in remission |
| ICD10 | opioid dependence | F11.22 | Opioid dependence with intoxication |
| ICD10 | opioid dependence | F11.222 | Opioid dependence with intoxication with perceptual disturbance |
| ICD10 | opioid dependence | F11.229 | Opioid dependence with intoxication, unspecified |
| ICD10 | opioid dependence | F11.24 | Opioid dependence with opioid-induced mood disorder |
| ICD10 | opioid dependence | F11.259 | Opioid dependence with opioid-induced psychotic disorder, unspecified |
| ICD10 | opioid dependence | F11.29 | Opioid dependence with unspecified opioid-induced disorder |
| ICD10 | opioid abuse | F11.122 | Opioid abuse with intoxication with perceptual disturbance |
| ICD10 | opioid abuse | F11.19 | Opioid abuse with unspecified opioid-induced disorder |
| ICD10 | opioid dependence | F11.220 | Opioid dependence with intoxication, uncomplicated |
| ICD10 | opioid dependence | F11.23 | Opioid dependence with withdrawal |
| ICD10 | opioid dependence | F11.251 | Opioid dependence with opioid-induced psychotic disorder with hallucinations |
| ICD10 | opioid dependence | F11.281 | Opioid dependence with opioid-induced sexual dysfunction |
| ICD10 | opioid dependence | F11.282 | Opioid dependence with opioid-induced sleep disorder |
| ICD10 | opioid dependence | F11.288 | Opioid dependence with other opioid-induced disorder |
| ICD10 | opioid abuse | F11.12 | Opioid abuse with intoxication |
| ICD10 | opioid abuse | F11.14 | Opioid abuse with opioid-induced mood disorder |
| ICD10 | opioid abuse | F11.151 | Opioid abuse with opioid-induced psychotic disorder with hallucinations |
| ICD10 | opioid abuse | F11.159 | Opioid abuse with opioid-induced psychotic disorder, unspecified |
| ICD10 | opioid abuse | F11.181 | Opioid abuse with opioid-induced sexual dysfunction |
| ICD10 | opioid abuse | F11.182 | Opioid abuse with opioid-induced sleep disorder |
| ICD10 | opioid dependence | F11.2 | Opioid dependence |
| ICD10 | opioid dependence | F11.20 | Opioid dependence, uncomplicated |
| ICD10 | opioid dependence | F11.221 | Opioid dependence with intoxication delirium |
| ICD10 | opioid dependence | F11.25 | Opioid dependence with opioid-induced psychotic disorder |
| ICD10 | opioid dependence | F11.250 | Opioid dependence with opioid-induced psychotic disorder with delusions |
| ICD10 | opioid dependence | F11.28 | Opioid dependence with other opioid-induced disorder |
| ICD10 | opioid poisoning | T40.0X1 | Poisoning by opium, accidental (unintentional) |
| ICD10 | opioid poisoning | T40.0X2 | Poisoning by opium, intentional self-harm |
| ICD10 | opioid poisoning | T40.0X3 | Poisoning by opium, assault |
| ICD10 | opioid poisoning | T40.0X4 | Poisoning by opium, undetermined |
| ICD10 | opioid poisoning | T40.1X1 | Poisoning by heroin, accidental (unintentional) |
| ICD10 | opioid poisoning | T40.1X2 | Poisoning by heroin, intentional self-harm |
| ICD10 | opioid poisoning | T40.1X3 | Poisoning by heroin, assault |
| ICD10 | opioid poisoning | T40.1X4 | Poisoning by heroin, undetermined |
| ICD10 | opioid poisoning | T40.2X1 | Poisoning by other opioids, accidental (unintentional) |
| ICD10 | opioid poisoning | T40.2X2 | Poisoning by other opioids, intentional self-harm |
| ICD10 | opioid poisoning | T40.2X3 | Poisoning by other opioids, assault |
| ICD10 | opioid poisoning | T40.2X4 | Poisoning by other opioids, undetermined |
| ICD10 | opioid poisoning | T40.3X1 | Poisoning by methadone, accidental (unintentional) |
| ICD10 | opioid poisoning | T40.3X2 | Poisoning by methadone, intentional self-harm |
| ICD10 | opioid poisoning | T40.3X3 | Poisoning by methadone, assault |
| ICD10 | opioid poisoning | T40.3X4 | Poisoning by methadone, undetermined |
| ICD10 | opioid poisoning | T40.411 | Poisoning by fentanyl or fentanyl analogs, accidental (unintentional) |
| ICD10 | opioid poisoning | T40.412 | Poisoning by fentanyl or fentanyl analogs, intentional self-harm |
| ICD10 | opioid poisoning | T40.413 | Poisoning by fentanyl or fentanyl analogs, assault |
| ICD10 | opioid poisoning | T40.414 | Poisoning by fentanyl or fentanyl analogs, undetermined |
| ICD10 | opioid poisoning | T40.421 | Poisoning by tramadol, accidental (unintentional) |
| ICD10 | opioid poisoning | T40.422 | Poisoning by tramadol, intentional self-harm |
| ICD10 | opioid poisoning | T40.423 | Poisoning by tramadol, assault |
| ICD10 | opioid poisoning | T40.424 | Poisoning by tramadol, undetermined |
| ICD10 | opioid poisoning | T40.491 | Poisoning by other synthetic narcotics, accidental (unintentional) |
| ICD10 | opioid poisoning | T40.492 | Poisoning by other synthetic narcotics, intentional self-harm |
| ICD10 | opioid poisoning | T40.493 | Poisoning by other synthetic narcotics, assault |
| ICD10 | opioid poisoning | T40.494 | Poisoning by other synthetic narcotics, undetermined |
| ICD10 | opioid poisoning | T40.601 | Poisoning by unspecified narcotics, accidental (unintentional) |
| ICD10 | opioid poisoning | T40.602 | Poisoning by unspecified narcotics, intentional self-harm |
| ICD10 | opioid poisoning | T40.603 | Poisoning by unspecified narcotics, assault |
| ICD10 | opioid poisoning | T40.604 | Poisoning by unspecified narcotics, undetermined |
| ICD10 | opioid poisoning | T40.691 | Poisoning by other narcotics, accidental (unintentional) |
| ICD10 | opioid poisoning | T40.692 | Poisoning by other narcotics, intentional self-harm |
| ICD10 | opioid poisoning | T40.693 | Poisoning by other narcotics, assault |
| ICD10 | opioid poisoning | T40.694 | Poisoning by other narcotics, undetermined |
