# Supplementary material for: Prediction of opioid-related outcomes in a medicaid surgical population: Evidence to guide postoperative opiate therapy and monitoring
Source: PLoS Comput Biol. 2023 Aug 14;19(8):e1011376. doi: 10.1371/journal.pcbi.1011376 (PMC10449152; doi:10.1371/journal.pcbi.1011376)
Supplement: S3 Table — (DOCX) [file pcbi.1011376.s003.docx]

## sTable 3: CCSR diagnosis categories for the 3 vulnerable populations

| **Population** | **CCSR categories** |
| --- | --- |
| Diabetes | END002, END003, END004, END005, END006 |
| Obesity | END009 |
| Depression | MBD002 |

## 
