# Supplementary material for: Prediction of opioid-related outcomes in a medicaid surgical population: Evidence to guide postoperative opiate therapy and monitoring
Source: PLoS Comput Biol. 2023 Aug 14;19(8):e1011376. doi: 10.1371/journal.pcbi.1011376 (PMC10449152; doi:10.1371/journal.pcbi.1011376)
Supplement: S4 Table — (DOCX) [file pcbi.1011376.s004.docx]

## sTable 4: Description of features

| **Demographics** | **Diagnosis** | **Procedures** | **Opioid prescriptions^(1)^** | **Non-opioid prescriptions** |
| --- | --- | --- | --- | --- |
| - Age - Gender - Rural-urban continuum code - Urban influence code | - 544 CCSR ICD-10-CM diagnosis categories (selected 227) | - Surgery type - 326 CCSR ICD-10-PCS procedure categories (selected 38) | - Opioid naive status - Cumulative daily MME^(2)^ - Discharge prescription daily MME - Number of opioid prescriptions - Number of unique prescribers - Longest continuous opioid use - Opioid type of discharge prescription - Number of standardized 30-day prescriptions - By opioid type^(3)^: - Cumulative daily MME - Number of prescriptions | - Number of prescriptions by medication type^(4)^ |

(1) Prescriptions (opioid and non-opioid) are counted during the 6-month period preceding surgery

(2) Morphine Milligram Equivalent (MME) of a prescription: quantity dispensed multiplied by strength (in milligrams) multiplied by a conversion factor. For a prescription, daily MME is the MME divided by the day's supply. Cumulative daily MME is the sum of daily MMEs of all prescriptions for a given patient.

(3) List of opioid types considered: codeine, hydrocodone, hydromorphone, morphine, oxycodone, tramadol

(4) List of medications considered: naltrexone, fluoxetine, buprenorphine, bupropion, dacomitinib, paroxetine, quinidine, acetaminophen, ibuprofen, diclofenac, ketorolac, naproxen, duloxetine, milnacipran, venlafaxin, gabapentin, pregabalin, amitriptalin, doxipen, imipramine, desipramine, nortryptaline, lidocaine, capsaicin.

## 
