# Supplementary material for: Prediction of opioid-related outcomes in a medicaid surgical population: Evidence to guide postoperative opiate therapy and monitoring
Source: PLoS Comput Biol. 2023 Aug 14;19(8):e1011376. doi: 10.1371/journal.pcbi.1011376 (PMC10449152; doi:10.1371/journal.pcbi.1011376)
Supplement: S5 Table — (DOCX) [file pcbi.1011376.s005.docx]

## sTable 5: Hyperparameters found by grid search with 5-fold cross-validation (Python 3.6.9)

| **Model** | **Hyperparameters for OAO** | **Hyperparameters for persistent opioid use** |
| --- | --- | --- |
| **Logistic Regression** | Default | Default |
| **Ridge** | C = 0.001 | C = 0.001 |
| **Lasso** | C = 0.01 | C = 0.05 |
| **ElasticNet** | C = 0.01  Mixing parameter = 0.6 | C = 0.01  Mixing parameter = 0.5 |
| **Random Forest** | Number of trees = 200 Maximum depth = 20 | Number of trees = 200 Maximum depth = 20 |
| **XGBoost** | Learning rate = 0.1  L2 regularization parameter = 1 | Learning rate = 0.1  L2 regularization parameter = 1 |
| **Deep Neural Net** | 6 dense layers with dimensions (128,128,128,128,32,8) and RELU activations  Dropout after each dense layer with probability 0.4.  Sigmoid output layer  Optimizer: Adam (learning rate = 0.0001) | 5 dense layers with dimensions (128,128,128,32,8) and RELU activations  Dropout after each dense layer with probability 0.4.  Sigmoid output layer  Optimizer: Adam (learning rate = 0.001) |
