# Supplementary material for: Prediction of opioid-related outcomes in a medicaid surgical population: Evidence to guide postoperative opiate therapy and monitoring
Source: PLoS Comput Biol. 2023 Aug 14;19(8):e1011376. doi: 10.1371/journal.pcbi.1011376 (PMC10449152; doi:10.1371/journal.pcbi.1011376)
Supplement: S6 Table — (DOCX) [file pcbi.1011376.s006.docx]

## sTable 6: Model performance metrics for opioid abuse/dependence/overdose prediction

| **Model** | **AUC** | **F1** | **Recall** | **Precision** |
| --- | --- | --- | --- | --- |
| **Logistic Regression** | 0.869 ± 0.013 | 0.55 ± 0.02 | 0.67 ± 0.04 | 0.46 ± 0.02 |
| **Ridge** | 0.870 ± 0.014 | 0.55 ± 0.02 | 0.67 ± 0.04 | 0.46 ± 0.02 |
| **Lasso** | 0.871 ± 0.014 | 0.55 ± 0.03 | 0.66 ± 0.04 | 0.47 ± 0.02 |
| **ElasticNet** | 0.871 ± 0.013 | 0.55 ± 0.03 | 0.66 ± 0.04 | 0.47 ± 0.02 |
| **Random Forest** | **0.877** ± 0.014 | **0.57** ± 0.01 | **0.69** ± 0.02 | **0.48** ± 0.02 |
| **XGBoost** | **0.877** ± 0.011 | 0.55 ± 0.02 | 0.68 ± 0.04 | 0.47 ± 0.01 |
| **Deep Neural Net** | 0.871 ± 0.013 | 0.55 ± 0.02 | 0.66 ± 0.03 | 0.47 ± 0.02 |
