# Supplementary material for: Prediction of opioid-related outcomes in a medicaid surgical population: Evidence to guide postoperative opiate therapy and monitoring
Source: PLoS Comput Biol. 2023 Aug 14;19(8):e1011376. doi: 10.1371/journal.pcbi.1011376 (PMC10449152; doi:10.1371/journal.pcbi.1011376)
Supplement: S7 Table — (DOCX) [file pcbi.1011376.s007.docx]

## sTable 7: Model performance metrics for persistent opioid use prediction

| **Model** | **AUC** | **F1** | **Recall** | **Precision** |
| --- | --- | --- | --- | --- |
| **Logistic Regression** | 0.814 ± 0.014 | 0.57 ± 0.03 | 0.49 ± 0.03 | **0.69** ± 0.02 |
| **Ridge** | 0.814 ± 0.013 | 0.58 ± 0.03 | **0.53** ± 0.03 | 0.66 ± 0.02 |
| **Lasso** | 0.814 ± 0.013 | **0.59** ± 0.03 | **0.53** ± 0.03 | 0.66 ± 0.02 |
| **ElasticNet** | 0.815 ± 0.013 | 0.58 ± 0.03 | 0.52 ± 0.03 | 0.67 ± 0.02 |
| **Random Forest** | 0.809 ± 0.012 | 0.58 ± 0.03 | 0.52 ± 0.04 | 0.66 ± 0.03 |
| **XGBoost** | **0.819** ± 0.010 | **0.59** ± 0.03 | 0.52 ± 0.04 | 0.68 ± 0.03 |
| **Deep Neural Net** | 0.809 ± 0.016 | 0.58 ± 0.03 | 0.52 ± 0.03 | 0.65 ± 0.02 |

# 
