# Supplementary figures and images for: Prediction of opioid-related outcomes in a medicaid surgical population: Evidence to guide postoperative opiate therapy and monitoring
Source: PLoS Comput Biol. 2023 Aug 14;19(8):e1011376. doi: 10.1371/journal.pcbi.1011376 (PMC10449152; doi:10.1371/journal.pcbi.1011376)

## sFig. 1: Flowchart for the study cohort


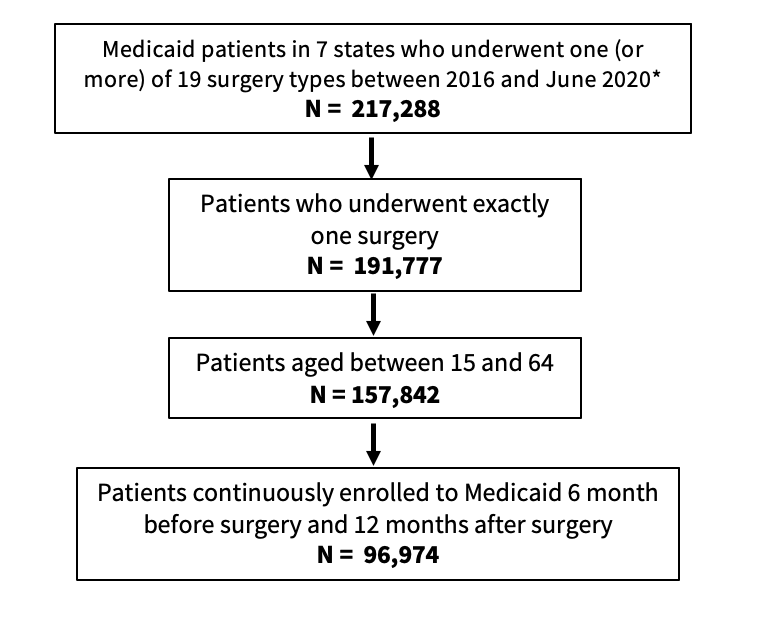

Supplement: S1 Fig — (DOCX) [file pcbi.1011376.s008.docx]

## sFig. 2: Precision-recall curve for random forest model

**
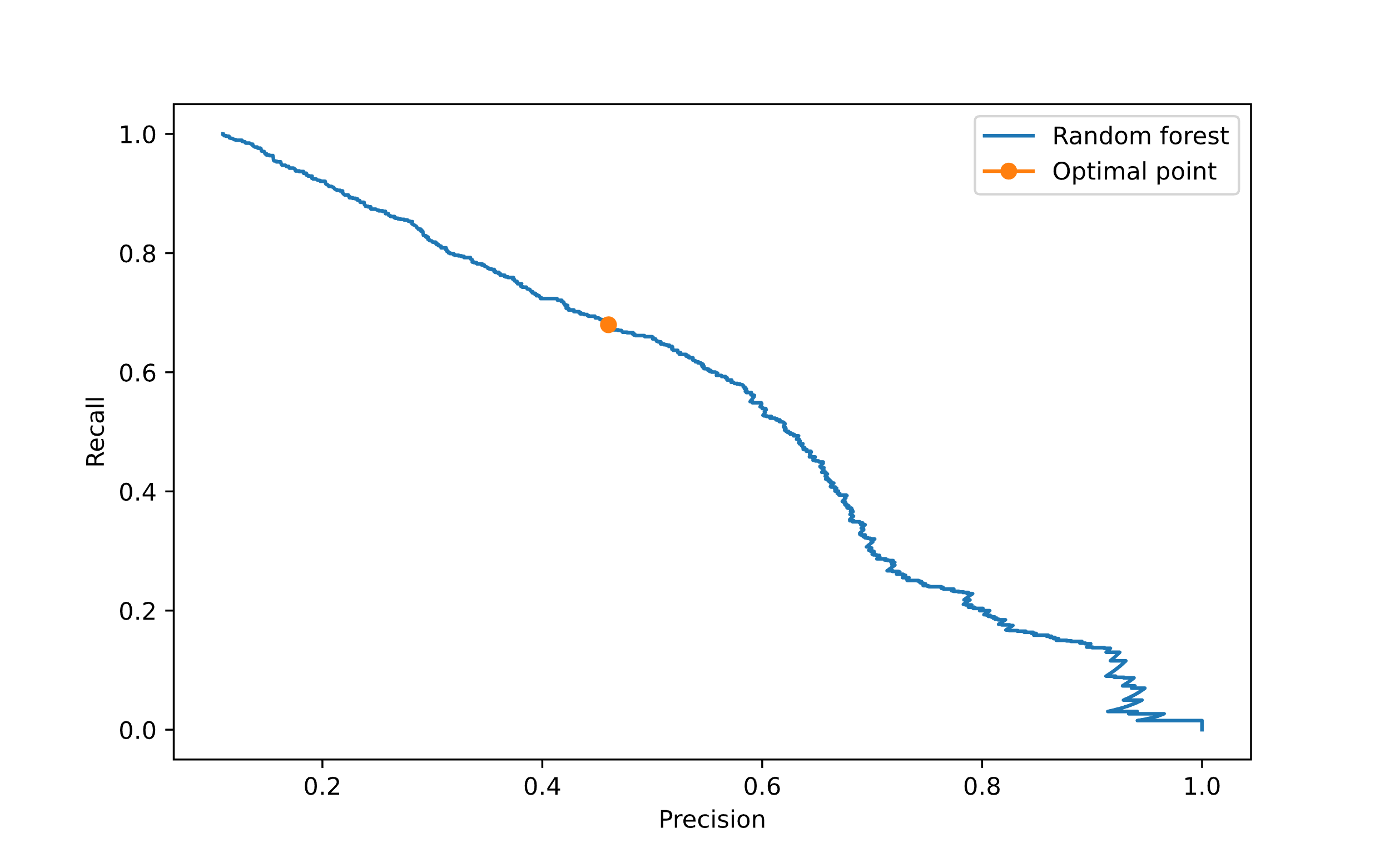
**

Supplement: S2 Fig — (DOCX) [file pcbi.1011376.s009.docx]
